# Prevalence and Clinical Phenotypes of Headache in Myelin Oligodendrocyte Glycoprotein Antibody‐Associated Disease: The MOGHEAD Study

**DOI:** 10.1155/prm/4241103

**Published:** 2026-10-06

**Authors:** Assunta Bianco, Matteo Lucchini, Costanza Sottani, Alessandra Cicia, Fabiana Cerulli, Gabriele Di Sante, Marco Bisurgi, Jacopo d’Angiò, Catello Vollono, Paolo Calabresi, Massimiliano Mirabella

**Affiliations:** ^1^ Centro di Ricerca per la Sclerosi Multipla “Anna Paola Batocchi” Catholic University of the Sacred Heart Faculty of Medicine and Surgery Largo A Gemelli 8 Rome Lazio IT 00168 Italy; ^2^ Neurology Fondazione Policlinico Universitario Agostino Gemelli IRCCS Largo A Gemelli 8 Rome IT 00168 Italy; ^3^ Section of Human Clinical and Forensic Anatomy University of Perugia Department of Medicine and Surgery P.le Severi n.1 Perugia Umbria IT 06132 Italy

**Keywords:** clinical phenotypes, migraine, MOGAD, pain, prevalence, symptoms

## Abstract

**Background:**

Headache associated with myelin oligodendrocyte glycoprotein antibody‐associated disease (MOGAD) is poorly characterized.

**Objective:**

To investigate the prevalence, clinical phenotypes, and longitudinal evolution of headache in MOGAD.

**Methods:**

In this single‐center retrospective cohort study based on prospectively collected clinical data, consecutive adult patients diagnosed with MOGAD between 2020 and 2024 were enrolled. Demographic, clinical, and radiological data were collected. Headaches were classified according to the International Classification of Headache Disorders. Patients underwent quarterly evaluations over 1 year. Pain severity was assessed using the Numeric Rating Scale at baseline and at the 1‐year visit, while headache‐related disability was assessed using the Migraine Disability Assessment at the 1‐year visit in patients reporting headache.

**Results:**

Twenty‐two patients were included (median age 35 years; 54.5% male). Headache occurred in 54.5% at onset and 68.2% after 1 year. Pre‐existing primary headache disorders were present in 54.5%. Headaches showed heterogeneous features, commonly retro‐orbital or unilateral frontal pain with pulsating or pressure‐like quality and associated photophobia/phonophobia. Migraine was the predominant phenotype at 1 year. Headache characteristics evolved over time in 73.7% of the cases, while 50% reported inadequate response to symptomatic therapy. Female sex, pre‐existing headache, and rituximab exposure were associated with headache persistence in univariable analyses.

**Conclusions:**

Headache is common in MOGAD, often pre‐existing, with heterogeneous phenotypes, although its clinical burden and evolution over time remain incompletely characterized. New‐onset retro‐orbital headache may represent a clinical red flag requiring further investigation.

**Trial Registration:** ClinicalTrials.gov identifier: NCT07479212

**Plain Language Summary**
• Headache is a common symptom in myelin oligodendrocyte glycoprotein antibody‐associated disease (MOGAD), but its frequency, characteristics, and evolution over time are not well understood. We followed adults with MOGAD for 1 year and found that headache was frequent both at disease onset and during follow‐up, often changed in its clinical features over time, and was inadequately controlled by symptomatic treatments in many patients. These findings show that headache is an important and persistent manifestation of MOGAD, highlighting the need for regular assessment and more effective management strategies to improve patient care.

## 1. Introduction

Myelin oligodendrocyte glycoprotein antibody‐associated disease (MOGAD) is a rare autoimmune inflammatory disorder of the central nervous system (CNS), characterized by IgG antibodies against myelin oligodendrocyte glycoprotein (MOG) on myelin sheaths [1–4]. Its prevalence may reach 2.5/100,000 [5, 6]. MOGAD affects children and young adults and usually presents a relapsing disease course, resulting in a substantial burden of lifelong disability. A positive serum MOG‐IgG test is a cornerstone for diagnosis. Unlike multiple sclerosis, for which a wide therapeutic armamentarium exists, treatment of MOGAD still relies largely on off‐label use of corticosteroids and immunosuppressive agents, underscoring a significant unmet clinical need [7, 8].

Pain is an increasingly relevant clinical feature in neuromyelitis optica spectrum disorders (NMOSDs) but is less well characterized in MOGAD [9, 10]. The mechanisms of pain in MOGAD remain unclear. A potential mechanism involves immune‐mediated oligodendrocyte damage, disrupting myelin integrity. Additionally, MOG has been shown to bind nerve growth factor (NGF), a key modulator of nociceptive pathways selectively expressed by unmyelinated sensory axons in the spinal cord and involved in activity‐dependent synaptic remodeling. Anti‐MOG–mediated disruption may impair NGF signaling, promoting maladaptive nociceptive fiber growth and nociceptive pain [11]. A small study showed that pain in patients with MOGAD is common, has a severe impact on the patients’ quality of life, and is insufficiently controlled by standard pharmacological treatments [9]. In another study, headache is frequently associated with optic neuritis and, in nearly 70% of the cases, precedes the visual deficit by several days, sometimes misdiagnosed as migraine [10]. Beyond orbital headache, patients with MOGAD may present with heterogeneous headache phenotypes, including migraine‐like and cervicogenic‐like presentations [12].

The International Classification of Headache Disorders (ICHD‐3) includes “headache attributed to noninfectious inflammatory intracranial disease” within secondary headaches. This category, with related subtypes, partly covers pain in multiple sclerosis, including trigeminal neuralgia and neuropathic pain syndromes [13]. However, headaches associated with MOGAD and NMOSD are not specifically categorized in ICHD‐3.

A recent review highlights pain as a frequent and clinically relevant symptom in MOGAD, affecting both pediatric and adult patients. It may present as headache, optic neuritis–related ocular pain, and facial or neuropathic pain, involving central and peripheral pathways. Pain can be acute or chronic and may precede disease onset or relapses. Management requires a multidimensional approach targeting both the autoimmune process and pain mechanisms, including pharmacological and integrative strategies, to optimize patient outcomes [14].

This study aims to characterize the prevalence and clinical phenotypes of headache in patients with MOGAD, focusing on headache at disease onset and its evolution over the first year following diagnosis.

## 2. Patients and Methods

### 2.1. Study Design

This is a single‐center retrospective cohort study based on prospectively collected clinical data. The study included all consecutive adult patients diagnosed with MOGAD according to the International MOGAD Panel criteria [1], between January 2020 and December 2024. All patients diagnosed with MOGAD had anti‐MOG antibodies that were positive on the live cell‐based assay test. Patients with MOGAD were routinely assessed every 3 months during the first year following disease onset. Headache‐related data, including information recorded in headache diaries, were collected during these clinical assessments and subsequently retrospectively analyzed for the purposes of the present study. The study was conducted in accordance with the Declaration of Helsinki and approved by the local Ethics Committee. Written informed consent was obtained from all participants.

### 2.2. Variables, Questionnaires, and Definitions

Demographic and clinical data included age, sex, clinical presentation at onset, magnetic resonance imaging (MRI) findings at disease onset, presence of oligoclonal bands, and treatments administered, including high‐dose intravenous corticosteroids and immunosuppressive therapies. Clinical presentation at onset was classified as isolated optic neuritis, transverse myelitis, acute disseminated encephalomyelitis (ADEM), brainstem or cerebellar features, or cerebral cortical encephalitis. ADEM is defined by acute, worsening over hours to days, polyfocal neurological deficits with encephalopathy (alteration in level of consciousness, profound irritability, and not related to postictal state) and by MRI features of multifocal T2 bright lesions often involving cerebral white and gray matter [1].

Clinical features of headache at disease onset included timing of onset, localization, pain quality, duration, and associated symptoms. A history of pre‐existing primary headache disorders was also collected, and headache phenotypes were classified according to the ICHD‐3 [13]. Potential changes in both pre‐existing and newly diagnosed headache patterns after MOGAD onset were assessed. Headache was assessed every 3 months over a 1‐year follow‐up period from the time of MOGAD diagnosis. Headache at the 1‐year follow‐up was defined as headache occurring during the preceding 3 months. Data on the evolution of headache, including its persistence, recurrence, resolution, or changes in phenotype over time, and symptomatic treatments used, were collected. Headache‐related pain was further assessed using structured questionnaires. The pain Numeric Rating Scale (NRS) [15], migraine disability assessment (MIDAS) [16], and Douleur Neuropathique 4 Questions (DN4) [17] were administered as appropriate to all patients who reported headache at the 1‐year follow‐up. MIDAS and DN4 questionnaires were not systematically administered at baseline. Resolution was defined as complete absence of headache during the 3 months preceding the 1‐year visit in a patient who had previously reported headache; improvement/worsening were defined as a decrease/increase in frequency and/or intensity (NRS) compared with the assessment at MOGAD onset or the previous quarterly visit. Due to low number of patients this is a hypothesis‐generating study.

Data on disease relapses, new neurological manifestations, and treatments initiated during follow‐up were also systematically collected.

### 2.3. Statistical Analysis

Continuous variables were summarized as median and interquartile range (IQR), while categorical variables were reported as percentages. To explore associations between baseline variables and the occurrence of headache, both at MOGAD onset and after 1‐year follow‐up, univariable logistic regression analyses were conducted. Multivariable analysis was performed on variable that were significantly associated at univariable analysis. Effect sizes were reported as odds ratios with 95% confidence intervals. All statistical analyses were performed using SPSS software (version 29; IBM Corporation, USA). A two‐tailed *p* value < 0.05 was considered statistically significant.

## 3. Results

Twenty‐two patients with MOGAD were included. The main characteristics at disease onset are summarized in Table 1. Median age at onset was 35 years (Q1–Q3: 28.6–39), with a slight male predominance (54.5%). At presentation, 31.8% had optic neuritis, 45.5% transverse myelitis, and 50% ADEM‐like symptoms. MRI most frequently showed spinal (63.6%) and supratentorial (59.1%) lesions. All patients with spinal cord lesions had both cervical and thoracic involvement. Oligoclonal bands were detected in 40.9%. Within 1 year, 68.2% experienced a clinical relapse. All patients received intravenous methylprednisolone (1 g/day for 5 days) followed by an oral taper (starting at 1 mg/kg over 3 months). The same regimen was also used at relapse. For maintenance, 12 patients received rituximab, 3 azathioprine, and 7 remained untreated due to a monophasic course. Twelve patients (54.5%) had a pre‐existing primary headache disorder before MOGAD onset, 12 patients (54.5%) reported headache at MOGAD onset, and 15 patients (68.2%) presented headache after 1‐year follow‐up. The longitudinal distribution and transitions of headache status are summarized in Figure 1.

**TABLE 1:** Characteristics of patients with MOGAD (*N* = 22).

Age at MOGAD onset (years)	35 (Q1–Q3: 28.6–39)
Male Sex	12 (54.5%)
Clinical presentation at onset^∗^	
Optic neuritis	7 (31.8%)
Transverse myelitis	10 (45.5%)
ADEM‐like symptoms	11 (50%)
MRI lesions at onset^∗^	
Optic nerve	5 (22.7%)
Spinal	14 (63.6%)
Supratentorial	13 (59.1%)
Infratentorial	7 (31.8%)
Oligoclonal bands	9 (40.9%)
Relapsing course	15 (68.2%)
Treatment	
Methylprednisolone therapy	22 (100%)
Rituximab	12 (54.5%)
Azathioprine	3 (13.6%)
Headache presentation	
History of primary headache before MOGAD onset	12 (54.5%)
Headache at MOGAD onset	12 (54.5%)
Headache after 1‐year follow‐up	15 (68.2%)

Abbreviations: ADEM, acute disseminated encephalomyelitis. MOGAD, myelin oligodendrocyte glycoprotein antibody‐associated disease. MRI, magnetic resonance imaging.

^∗^More than one clinical presentation and multiple sites of brain lesions may be present in the same patient.

**FIGURE 1:**
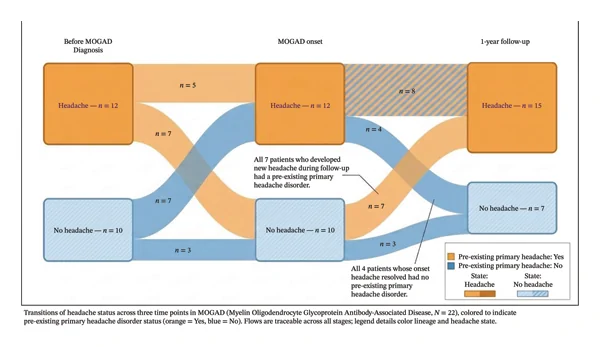
Transitions of headache status across three time points in myelin oligodendrocyte glycoprotein antibody‐associated disease (MOGAD, *n* = 22), colored to indicate pre‐existing primary headache disorder status (orange = yes, blue = no). Flows are traceable across all stages; legend details color lineage and headache state. Sankey diagram illustrating the distribution and transitions of headache status across three time points in patients with MOGAD (*N* = 22): before MOGAD diagnosis, at MOGAD onset, and at 1‐year follow‐up. Node fill indicates headache state at each time point (solid = headache; hatched = no headache), while flow color indicates the pre‐existing primary headache disorder status of each patient subgroup, carried through from baseline (orange = pre‐existing primary headache disorder present; blue = absent), allowing the trajectory of each subgroup to be traced across all three time points. Annotations highlight two key findings: all seven patients who developed new headache during follow‐up had a pre‐existing primary headache disorder, whereas all four patients whose onset headache resolved by 1‐year follow‐up had no pre‐existing primary headache disorder. MOGAD: myelin oligodendrocyte glycoprotein antibody‐associated disease.

A history of primary headache disorder was present in 12 patients (54.5%) of patients with MOGAD (Table 2). The median age at primary headache onset was 20 years (Q1–Q3: 14.5–23.5). Tension‐type headache (TTH) was the most frequent diagnosis (41.7%), followed by migraine with or without aura (33.3%), while 25% of patients reported both headache types. Median monthly frequency of headache episodes was 0.5 (Q1–Q3: 0–1). The most common headache locations were the unilateral and bilateral frontal regions (41.7% each); notably, no patients reported retro‐orbital headache as part of their primary headache phenotype. Pain quality was most frequently described as dull or pressure‐like (66.7%), followed by a pulsating quality (41.7%). Headache‐associated symptoms were present in 50% of the patients, and 58.3% reported identifiable triggers, including cold exposure, wind, menstruation, bright light, and stress. No patients had received prophylactic treatment for headache episodes.

**TABLE 2:** Characteristics of patients with headache.

	Primary headache before MOGAD onset (*n* = 12)	At MOGAD onset (*n* = 12)	At 1‐year follow‐up (*n* = 15)
Headache type			
Tension‐type	5 (41.7%)	Not applicable	4 (26.7%)
Migraine	4 (33.3%)		8 (53.3%)
Both types	3 (25%)		3 (20%)
Monthly frequency of headache episodes	0.5 (Q1–Q3L: 0–1)	Not applicable	0.5 (Q1–Q3: 0–2)
Headache locations^∗^			
Retro‐orbital	0	5 (41.7%)	1 (6.6%)
Frontal unilateral	5 (41.7%)	5 (41.7%)	6 (40%)
Frontal bilateral	5 (41.7%)	2 (16.7%)	3 (20%)
Parietal	2 (16.7%)	2 (16.7%)	2 (13.3%)
Holocranial	0	1 (8.3%)	1 (6.6%)
Occipital	2 (16.7%)	1 (8.3%)	2 (13.3%)
Cervical	0	1 (8.3%)	1 (6.6%)
Pain quality^∗^			
Dull or pressure‐like pain	8 (66.7%)	6 (50%)	7 (46.7%)
Pulsating pain	5 (41.7%)	5 (41.7%)	8 (53.3%)
Constrictive pain	1 (8.3%)	2 (16.7%)	2 (13.3%)
Headache‐associated symptoms	6 (50%)	7 (58.3%)	8 (53.3%)

^∗^More than one headache location and quality of pain may be present in the same patient.

Among 12 patients with headache at MOGAD onset, the median duration of headache episodes was 144 h (Q1–Q3: 60–240). Headache at disease onset was reported in 57.1% of the patients presenting with optic neuritis, 60% of those with transverse myelitis, and 45.4% of the patients with ADEM‐like symptoms. The most common headache locations were retro‐orbital and unilateral frontal regions (41.7% each). Pain quality was most commonly described as dull or pressure‐like (50%), followed by a pulsating quality (41.7%). Head pain was lateralized in 50% of the cases. Headache‐associated symptoms were present in 58.3% of the patients, most frequently photophobia, phonophobia, and vertigo (Table 2). Seven patients reported headache at MOGAD onset without a previous history of primary headache disorder. Of these, 71% were male, and the median age was 30 years (Q1–Q3: 27–37). The median headache duration was 168 h (Q1–Q3: 60–216). Headache localization included retro‐orbital (57%), unilateral frontal (57%), bilateral frontal (28%), and parietal (14%) regions. Lateralization of pain was reported by 57% of the patients. Pain was described as pulsating (42%), pressing (28%), and constrictive (28%). Overall, 57% of the patients reported associated symptoms, including photophobia, visual impairment, and vertigo. In the five patients with both a primary headache disorder and headache at MOGAD onset, the latter differed from their usual primary headache in terms of location, pain quality, associated symptoms, and triggers.

After one year of follow‐up, 15 patients (68.2%) reported headache. Of these, 8 had already experienced headache at MOGAD onset, whereas the remaining 7 developed it during follow‐up, but all of them had a previous diagnosis of primary headache. Conversely, headache resolved in 4 patients who presented with it at MOGAD onset; all had no prior diagnosis of primary headache (Figure 1). Migraine was the most frequent diagnosis (53.3%), followed by TTH (26.7%), while 20% of the patients reported both headache types (Table 2). The median monthly frequency of headache episodes was 0.5 (Q1–Q3: 0–2). Fourteen patients (93.3%) used one or more symptomatic treatments for headache (64.3% nonsteroidal anti‐inflammatory drugs, mainly ibuprofen, and 42.8% acetaminophen), but 50% reported suboptimal response. Specifically, a poor response was observed in 60% of the patients who took acetaminophen and in 11% of the patients who took nonsteroidal anti‐inflammatory drugs. No patient was prescribed triptans during the first year following MOGAD onset. Structured questionnaires were administered to 22 patients at the 1‐year follow‐up. Median NRS score was 6 (IQR 3), median MIDAS score was 3 (IQR 10), while DN4 was administered in a few patients due to the pain’s phenotype. Changes in headache pattern, in terms of frequency and/or intensity, were observed in 73.7% of the patients. Headache resolved in 21% of the patients, improved in 42.1% of the patients, was stable in 31.6%, and worsened in 5.3% (*n* = 19). A change in headache phenotype was observed in 5.3% of the patients; this change occurred in a patient whose overall headache pattern was classified as stable. None of the patients fulfilled the ICHD‐3 criteria for a chronic headache disorder, either before MOGAD onset or at the 1‐year follow‐up. Accordingly, no patient met the criteria for preventive pharmacological treatment of headache during the study period, and no prophylactic treatment was prescribed.

The results of the univariate analysis investigating predictors of headache at MOGAD onset are shown in Table 3. No baseline factors were found to be predictive of headache occurrence. Conversely, female sex (OR: 12.6, 95% CIs: 1.2–133.9, *p* = 0.04), pre‐existing primary headache (OR: 25.7, 95% CIs: 2.2–298.5, *p* = 0.01), and rituximab treatment (OR: 7.5, 95% CIs: 1.1–54.1, *p* = 0.05) were predictive for the occurrence of headache at 1‐year follow‐up. These associations did not retain statistical significance in the multivariable model, likely reflecting overfitting given the limited number of events (Table 4).

**TABLE 3:** Predictors of headache at MOGAD onset.

Variable	Odds ratio (95% CIs)	*p* value
Age	0.9 (0.8–1.0)	0.16
Female sex	1.5 (0.3–8.2)	0.64
Pre‐existing primary headache	0.3 (0.1–1.8)	0.19
Optic neuritis at onset	1.2 (0.2–7.1)	0.87
Transverse myelitis at onset	1.5 (0.3–8.2)	0.64
ADEM‐like at onset	0.5 (0.1–3.6)	0.46
Supratentorial lesions	2.1 (0.4–11.6)	0.39

Abbreviation: 95% CIs, 95% confidence intervals.

**TABLE 4:** Predictors of headache at 1‐year follow‐up.

Variable	Univariate analysis		Multivariate analysis	
	Odds ratio (95% CIs)	*p* value	Odds ratio (95% CIs)	*p* value
Age	1.0 (0.9–1.1)	0.90		
Female sex	12.6 (1.2–133.9)	0.04	4.7 (0.3–75.1)	0.28
Pre‐existing primary headache	25.7 (2.2–298.5)	0.01	11.3 (0.7–179.3)	0.09
Headache at MOGAD onset	0.6 (0.1–3.5)	0.57		
Optic neuritis at onset	5.3 (0.5–54.9)	0.17		
Transverse myelitis at onset	0.8 (0.1–4.3)	0.75		
ADEM‐like at onset	0.3 (0.1–2.2)	0.23		
Supratentorial lesions	1.0 (0.2–5.7)	1.0		
Rituximab treatment	7.5 (1.1–54.1)	0.05	2.4 (0.2–35.8)	0.52

Abbreviation: 95% CIs, 95% confidence intervals.

Regarding the temporal relationship between headache and clinical relapses, among patients with a relapsing disease course (*n* = 15), 53% presented with headache at MOGAD onset. Headache preceded the onset of the first clinical manifestation in only two patients, both of whom reported retro‐orbital pain followed by optic neuritis. During the 1‐year follow‐up, headache recurred in 80% of patients. However, in only four patients did headache precede a subsequent clinical relapse; in all four cases, the headache was characterized by retro‐orbital pain and was followed by optic neuritis. In the remaining patients, headache recurrence was not temporally associated with a subsequent clinical relapse. The available data did not allow us to reliably determine whether headache consistently reappeared following clinical relapses.

## 4. Discussion

In our single‐center cohort of patients with MOGAD, the prevalence of headache was 54.5% at disease onset and 68.2% after 1 year of follow‐up. Data available in the literature for comparison are limited. In the global aggregate review by Trewin et al. (*n* = 4699), headache specifically associated with optic neuritis was reported in 35% of the adult patients although this estimate was derived from only 5 studies (*n* = 65) and is not directly comparable with our cohort‐wide headache prevalence [6]. Similarly, in the prospective cohort by Asseyer et al., the prevalence of headache was 49.6% among 129 patients with MOGAD‐related optic neuritis [10]. These studies did not include a longitudinal assessment of headache. Our findings should also be interpreted in the context of the high background prevalence of headache in both CNS inflammatory disorders and the general population. In a recent systematic review and meta‐analysis, Gklinos et al. reported a pooled prevalence of 58% for headache disorders among patients with multiple sclerosis, with migraine and TTH accounting for 30% and 19%, respectively [18]. Similarly, a large meta‐analysis of population‐based individual participant data from 41,614 adults in 17 countries reported a 1‐year prevalence of any headache of 65.5%, including 23.5% for migraine and 33.2% for TTH [19]. Therefore, although headache was common in our MOGAD cohort, particularly at the 1‐year follow‐up, its prevalence cannot be interpreted as evidence of a disease‐specific association in the absence of a control group.

Our findings do not allow us to establish whether headache in MOGAD represents an acute manifestation of the disease, a persistent or recurrent secondary headache related to MOGAD, a worsening of a pre‐existing primary headache disorder, or a new‐onset primary headache triggered by the disease. The longitudinal assessment provides some insights into these possible patterns. Although headache was present in more than half of patients at disease onset, its persistence during the first year suggests that, in some patients, it may not be limited to the acute phase of MOGAD. Conversely, the recurrence or persistence of headache during follow‐up cannot, by itself, establish a secondary relationship with MOGAD, particularly in patients with a pre‐existing primary headache disorder, in whom changes in headache frequency or phenotype may occur independently of disease activity. Similarly, in patients without a previous history of primary headache, the emergence of a migraine‐like phenotype during follow‐up may reflect either a secondary headache with migrainous features or a new‐onset primary headache; our study was not designed to distinguish between these possibilities.

During the first year of follow‐up, 73.7% of the patients experienced a change in headache pattern. While TTH was the most frequent phenotype before MOGAD onset (41.7%), a migraine‐like phenotype became predominant at 1 year (53.3%). It would be interesting to explore whether this “migrainization” is connected to the suggested impairment of NGF signaling [11]. The possible link between NGF signaling and nociceptive‐fiber mechanisms represents a hypothesis that should be tested in future studies incorporating biomarker or neurophysiological data. It is important to highlight that, in our cohort, none of patients with migraine received triptan treatment. In many patients, the recent diagnosis of MOGAD and the start of immunosuppressive therapy made them reluctant to change their previous treatment approach for headache.

During the first year after MOGAD onset, headache had a limited impact on daily activities, as reflected by MIDAS scores, but was characterized by moderate intensity according to NRS and a suboptimal response to symptomatic pharmacological treatment. Overall, headache represents a symptom that should not be overlooked in clinical practice.

With regard to the assessment of factors predicting headache occurrence, no significant predictors were identified at baseline, whereas female sex, pre‐existing primary headache, and rituximab treatment were associated with headache occurrence at the 1‐year follow‐up in the univariable analysis. In our view, the association between rituximab exposure and headache most likely reflects indication bias, as rituximab was preferentially prescribed to patients with a more severe or relapsing disease course, rather than a direct pharmacological effect. However, a potential contribution of complement‐ or cytokine‐mediated mechanisms cannot be excluded.

An interesting topic that has emerged in the literature in this field concerns the association between headache and optic neuritis. In the previously mentioned cohort by Asseyer et al., prodromal headache is a common symptom; it precedes visual loss by a few days and has a characteristic retro‐orbital location with possible unilateral frontal extension [10]. Vilaseca et al. described eight cases of optic neuritis associated with MOGAD preceded by painful amaurosis fugax, characterized by brief, transient (< 1 h) episodes of unilateral visual loss associated with pain. Painful amaurosis fugax preceded the onset of optic neuritis by a few days [20]. Both authors caution that this condition may be mistaken for migraine‐related pain, potentially leading to delayed diagnosis. In our cohort, 57.1% of the patients presenting with optic neuritis reported headache, typically with a retro‐orbital location. Notably, no patients reported retro‐orbital headache as part of their primary headache phenotype.

Study limitations include the relatively small sample size, which may limit the statistical power and generalizability of the findings. Given the low event‐to‐variable ratio, the multivariable model is likely overfitted; the loss of statistical significance for female sex and pre‐existing headache should not be interpreted as evidence against these associations but as a consequence of limited statistical power. However, given the rarity of MOGAD, this constraint is difficult to overcome in single‐center studies and can only be adequately addressed through multicenter collaborations or large prospective registry‐based cohorts. Structured headache questionnaires were not systematically available at baseline; therefore, a longitudinal comparison of headache severity, disability, and neuropathic features between baseline and the 1‐year follow‐up was not possible.

## 5. Conclusions

In conclusion, headache is a common feature of MOGAD, occurring in more than half of the patients at disease onset and remaining frequent during the first year of follow‐up. The higher prevalence at 1 year was largely driven by patients with pre‐existing primary headache disorders and should not be interpreted as evidence of a MOGAD‐related increase in headache occurrence. Headache showed heterogeneous phenotypes and, in some patients, a persistent or recurrent course, with limited functional impact but a suboptimal response to symptomatic treatment. New‐onset retro‐orbital headache, particularly when differing from a patient’s previous headache pattern, may represent a clinical red flag for MOGAD. The nature and pathophysiological mechanisms of headache in MOGAD remain uncertain and warrant confirmation in larger, prospective multicenter studies.

**Nomenclature**
ADEM: Acute disseminated encephalomyelitis
CNS: Central nervous system
DN4: Douleur neuropathique 4 questions
ICHD‐3: International classification of headache disorders, 3rd edition
IgG: Immunoglobulin G
MIDAS: Migraine disability assessment
MOG: Myelin oligodendrocyte glycoprotein
MOGAD: Myelin oligodendrocyte glycoprotein antibody‐associated disease
MRI: Magnetic resonance imaging
NGF: Nerve growth factor
NMOSD: Neuromyelitis optica spectrum disorders
NRS: Numeric Rating Scale
TTH: Tension‐type headache

## Funding

This study did not receive any funding.

Open access publishing facilitated by Universita Cattolica del Sacro Cuore, as part of the Wiley ‐ CRUI‐CARE agreement.

## Conflicts of Interest

Assunta Bianco has received honoraria for speaking, advisory board from Bio gen, Novartis, Merck Serono, Roche, Sanofi Genzyme, Bristol‐Myers Squibb, Alexion unrelated to the present work. Matteo Lucchini has received hon oraria for speaking, advisory board from Biogen, Novartis, Merck Serono, Roche, Sanofi Genzyme, Bristol‐Myers Squibb, Alexion unrelated to the present work. Alessandra Cicia has received travel grant from Novartis, Merck Serono, Alexion unrelated to the present work. Massimiliano Mirabella has received honoraria for speaking, advisory board/consulting from Biogen, Novartis, Merck Serono, Roche, Sanofi Genzyme, Janssen, Bristol‐Myers Squibb, Viatris, Alexion unrelated to the present work. The other authors declare no conflicts of interest.

## Data Availability

The data that support the findings of this study are available from the corresponding author upon reasonable request.

## References

[1] Banwell B. , Bennett J. L. , Marignier R. et al., Diagnosis of Myelin Oligodendrocyte Glycoprotein Antibody-Associated Disease: International MOGAD Panel Proposed Criteria, Lancet Neurol. (2023) 22, no. 3, 268–282, 10.1016/S1474-4422(22)00431-8. 36706773

[2] Marignier R. , Hacohen Y. , Cobo-Calvo A. et al., Myelin-Oligodendrocyte Glycoprotein Antibody-Associated Disease, Lancet Neurol. (2021) 20, no. 9, 762–772, Erratum in: Lancet Neurol. 2021 Oct;20(10):e6. doi: 10.1016/S1474-4422(21)00290-8. Erratum in: Lancet Neurol. 2022 Jan;21(1):e1. doi: 10.1016/S1474-4422(21)00433-6 10.1016/S1474-4422(21)00218-0. 34418402

[3] Juryńczyk M. , Jacob A. , Fujihara K. , and Palace J. , Myelin Oligodendrocyte Glycoprotein (MOG) antibody-associated Disease: Practical Considerations, Practical Neurology. (2019) 19, no. 3, 187–195, 10.1136/practneurol-2017-001787. 30530724

[4] Dos Passos G. R. , Oliveira L. M. , da Costa B. K. et al., MOG-IgG-Associated Optic Neuritis, Encephalitis, and Myelitis: Lessons Learned From Neuromyelitis Optica Spectrum Disorder, Frontiers in Neurology. (2018) 9, 10.3389/fneur.2018.00217. PMC5893792 29670575

[5] Hor J. Y. and Fujihara K. , Epidemiology of Myelin Oligodendrocyte Glycoprotein Antibody-Associated Disease: A Review of Prevalence and Incidence Worldwide, Frontiers in Neurology. (2023) 14, 10.3389/fneur.2023.1260358. PMC10542411 37789888

[6] Trewin B. P. , Brilot F. , Reddel S. W. , Dale R. C. , and Ramanathan S. , MOGAD: a Comprehensive Review of Clinicoradiological Features, Therapy and Outcomes in 4699 Patients Globally, Autoimmunity Reviews. (2025) 24, no. 1, 10.1016/j.autrev.2024.103693. 39577549

[7] Cacciaguerra L. and Flanagan E. P. , Updates in NMOSD and MOGAD Diagnosis and Treatment: A Tale of Two Central Nervous System Autoimmune Inflammatory Disorders, Neurologic Clinics. (2024) 42, no. 1, 77–114, 10.1016/j.ncl.2023.06.009. PMC10658081 37980124

[8] de Mol C. L. , Wong Y. , van Pelt E. D. et al., The Clinical Spectrum and Incidence of Anti-MOG-Associated Acquired Demyelinating Syndromes in Children and Adults, Multiple Sclerosis. (2020) 26, no. 7, 806–814, 10.1177/1352458519845112. PMC7294530 31094288

[9] Asseyer S. , Schmidt F. , Chien C. et al., Pain in AQP4-IgG-Positive and MOG-IgG-positive Neuromyelitis Optica Spectrum Disorders, Mult Scler J Exp Transl Clin. (2018) 4, no. 3, 10.1177/2055217318796684. PMC6117869 30186620

[10] Asseyer S. , Hamblin J. , Messina S. et al., Prodromal Headache in MOG-Antibody Positive Optic Neuritis, Multiple Sclerosis and Related Disorders. (2020) 40, 10.1016/j.msard.2020.101965. 32062443

[11] von Büdingen H. C. , Mei F. , Greenfield A. et al., The Myelin Oligodendrocyte Glycoprotein Directly Binds Nerve Growth Factor to Modulate Central Axon Circuitry, The Journal of Cell Biology. (2015) 210, no. 6, 891–898, 10.1083/jcb.201504106. PMC4576870 26347141

[12] Asseyer S. , Cooper G. , and Paul F. , Pain in NMOSD and MOGAD: A Systematic Literature Review of Pathophysiology, Symptoms, and Current Treatment Strategies, Frontiers in Neurology. (2020) 11, 10.3389/fneur.2020.00778. PMC7812141 33473247

[13] Headache Classification Committee of the International Headache Society (IHS) the International Classification of Headache Disorders, Cephalalgia. (2018) 38, 3rd edition, no. 1, 1–211, 10.1177/0333102417738202. 29368949

[14] Diaz P. , Nealon N. E. , and Kaunzner U. W. , Pain and Headache in Myelin Oligodendrocyte Glycoprotein Antibody-Associated Disease, Current Pain and Headache Reports. (2025) 29, no. 1, 10.1007/s11916-024-01322-7. 39878785

[15] Jensen M. P. , Karoly P. , and Braver S. , The Measurement of Clinical Pain Intensity: A Comparison of Six Methods, Pain. (1986) 27, no. 1, 117–126, 10.1016/0304-3959(86)90228-9. 3785962

[16] Stewart W. F. , Lipton R. B. , Dowson A. J. , and Sawyer J. , Development and Testing of the Migraine Disability Assessment (MIDAS) Questionnaire to Assess Headache-Related Disability, Neurology. (2001) 56, no. 6 Suppl 1, S20–S28, 10.1212/wnl.56.suppl_1.s20. 11294956

[17] Abolkhair A. B. , El-Kabbani A. O. , Al-Mulhem A. et al., Psychometric and Accuracy Comparison of Three Commonly Used Questionnaires for the Diagnosis of Neuropathic Pain, Saudi Journal of Anaesthesia. (2021) 15, no. 4, 409–418, 10.4103/sja.sja_352_21. PMC8477763 34658728

[18] Gklinos P. and Mitsikostas D. D. , Headache Disorders in Multiple Sclerosis: Is There an Association? A Systematic Review and meta-analysis, Multiple Sclerosis and Related Disorders. (2024) 85, 10.1016/j.msard.2024.105536. 38489946

[19] Husøy A. K. , Yu S. , Liu R. et al., The Global Prevalence of Headache Disorders of public-health Importance: A Meta-Analysis of Population-Based Individual Participant Data From 41,614 Adults From 17 Countries, The Journal of Headache and Pain. (2025) 26, no. 1, 10.1186/s10194-025-02142-9. PMC12502191 41057756

[20] Vilaseca A. , Syc-Mazurek S. B. , Vorasoot N. et al., Amaurosis Fugax Dolorosa: A Prodromal Syndrome in Optic Neuritis Associated With Myelin Oligodendrocyte Glycoprotein Antibody-Associated Disease, Neurol Neuroimmunol Neuroinflamm. (2025) 12, no. 6, Erratum in: Neurol Neuroimmunol Neuroinflamm. 2026 Jan;13(1):e200526. doi: 10.1212/NXI.0000000000200526 10.1212/NXI.0000000000200471. PMC12624419 41248449

